# Predicting Academic Performance Among Female Undergraduate Medical and Health Sciences Students Through Sociodemographic, Socioeconomic, and Nutritional Factors

**DOI:** 10.7759/cureus.100359

**Published:** 2025-12-29

**Authors:** Sahiba Hakro, Mehrun Nisa Soomro, Fiza Keerio, Sher M Chandio, Fareeha Hassan

**Affiliations:** 1 Public Health, Ziauddin University, Karachi, PAK; 2 Public Health, Peoples University of Medical and Health Sciences for Women, Shaheed Benazirabad, PAK; 3 Community Medicine, Peoples University of Medical and Health Sciences for Women, Shaheed Benazirabad, PAK; 4 Public Health, Jinnah Sindh Medical University, Karachi, PAK

**Keywords:** academic performance, female undergraduates, health sciences students, medical students, nutritional factors, sociodemographic factors, socioeconomic status

## Abstract

Introduction

Education plays a crucial role in improving individual well-being and socioeconomic development. Poor academic performance is often associated with adverse socioeconomic and psychosocial outcomes. Numerous studies have highlighted the effects of sociodemographic, socioeconomic, and nutritional factors on academic performance. However, these studies have been limited to school students only, leaving university students understudied.

Objective

To predict academic performance through sociodemographic, socioeconomic, and nutritional factors among female undergraduate medical and health sciences students.

Materials and methods

This cross-sectional study was conducted on 400 female undergraduate medical and health sciences students selected using stratified systematic random sampling. Data were collected through a self-administered questionnaire. Nutritional status was assessed with body mass index (BMI), and academic performance was assessed with mean aggregate marks. Chi-square test was used to assess the associations. Binary logistic regression was used to estimate odds ratios (OR) with 95% confidence intervals (CI). Variables showing significance in binary logistic regression were included in multiple logistic regression model, and adjusted odds ratios (AOR) and their 95% CI were reported.

Results

The mean age of participants was 21.5±1.5 years. Fifty percent students had normal weight with a mean BMI of 21.8±5.7 kg/m² and 76.8% students demonstrated good academic performance. On bivariate analysis academic performance was significantly higher among Bachelor of Medicine, Bachelor of Surgery (MBBS) students (OR=6.23, 95% CI: 3-13.03), and Bachelor of Science in Public Health (BSPH) students (OR=2.91, 95% CI: 1.21-7.27), as well as the fourth (OR=2.84, 95% CI: 1.54-5.37) and fifth year students (OR=7.15, 95% CI: 2.9-21.7). Performance was also significantly associated with upper-middle-class, mixed diet, three or more meals per day, and daily breakfast. In the multivariate model, field and year of study emerged as key predictors. The model revealed 79.3% accuracy with a positive predictive value of 80.8%.

Conclusion

Academic performance is a multifaceted outcome influenced by academic-related factors, particularly academic progression and field of study. Nutritional and socioeconomic factors had a limited independent effect.

## Introduction

Education enhances livelihoods, health, and promotes social stability. At the individual level, it is associated with a better quality of life through enhanced productivity, as educated individuals have greater social and economic opportunities. On a broader scale, it builds talented and well-informed human capital, which is considered a driving force for economic development [1]. Academic performance refers to the extent to which an institution, student, or teacher has achieved their goals [2].

Academically strong students tend to have higher self-confidence and self-esteem, lower anxiety levels and depression, better social orientation, and a lower likelihood of being involved in substance use such as khat and alcohol [1]. Poor academic performance often leads to poverty, unemployment, promiscuity, drug use, illegal activities, homelessness, social isolation, dependency, and inadequate health insurance [3]. Academic performance is a multifaceted outcome affected by various interlinked factors, including psychological traits, study settings and environment, extracurricular participation, communication skills, and even physical health [4].

Sociodemographic characteristics significantly shape the academic outcome of the students by providing a supportive environment [5]. On the other hand, socioeconomic status is a key measure of an individual’s overall prestige in society. It is an extensively researched construct in social sciences and encompasses dimensions like parental occupation, education, income, access to opportunities and resources [6,7]. Research has consistently demonstrated a strong association between socioeconomic status and academic success [7].

Medical and health sciences students frequently experience hectic schedules and high academic demands, which leave little room for prioritizing their own nutrition and health. This contributes to unhealthy eating behaviours such as skipping meals and eating unhealthy snacks, leading to elevated stress levels [8]. Medical students often under-eat or overeat, leading to nutrient deficiencies that can affect their educational performance [9]. Given the well-documented influence of nutritional status on students' cognitive function, focus, and general academic accomplishment, the relationship between academic performance and nutrition has received more attention recently [10].

Most studies on academic performance are concentrated on primary and secondary level students leaving gap for female university students. The objective of this study is to predict academic performance through sociodemographic, socioeconomic, and nutritional factors among female undergraduate medical and health sciences students.

## Materials and methods

Study design, duration, and settings

This cross-sectional study was conducted from March 21, 2023, to August 16, 2023, on female undergraduate medical and health sciences students at Peoples University of Medical and Health Sciences for Women (PUMHSW), Shaheed Benazirabad, Sindh, Pakistan.

Sample size, technique and selection

The sample size was calculated using Cochran's formula n=z^2^ x p (1-p)/d^2^ [11], assuming a 70.5% prevalence (p=0.705) for good academic performance among health sciences students, as reported in a previous Ethiopian study, with a z-value of 1.96 corresponding to a 95% confidence level and a 5% margin of error (d=0.05) [12]. Although the minimum required sample size was 321, we recruited 400 participants using stratified systematic random sampling to ensure proportional representation across fields. Consenting female undergraduate medical and health sciences students aged 18 years and above who appeared in their last regular annual or semester exams were included in the study. Nonconsenting students, first-year students, and those who didn’t appear in their last regular or semester examinations were excluded from the study.

Study instrument and validation

The study instrument was a self-administered questionnaire consisting of 22 items divided into four sections (Appendix A). The first sections comprised five items assessing sociodemographic characteristics. The second section comprised five items assessing socioeconomic status. The third section comprised nine items related to nutritional information. The last section comprised three items to examine academic performance. The questionnaire was developed by the authors themselves after careful review of the relevant literature. Its content validity was assessed by six experts. The overall content validity index was 0.93, reflecting excellent validity. A pilot study was conducted on 20 students to assess the face validity, clarity, and administration.

Father's occupation was classified into six categories depending upon the nature of the profession: government employee, private employee, doctor, businessman, teacher, and others. Mother's occupation was classified into two categories: unemployed and employed. Parental education was classified based on years of formal education and stratified into four categories: illiterate (no formal education), primary (one to five years), secondary and higher secondary (six to 12 years), and graduation (≥14 years). Parental income was measured on a monthly basis and was classified into four categories: lower class (<50,000 Pakistani rupee or PKR), lower-middle class (50,000-100,000 PKR), upper-middle class (100,001-200,000 PKR), and upper class (>200,000 PKR).

Nutritional status was assessed with BMI and classified into three categories: underweight (<18.5 kg/m^2^, normal weight (18.5-24.9 kg/m^2^), and overweight (≥25.0 kg/m^2^). The frequency of breakfast, lunch, and dinner was assessed based on weekly consumption and classified into three categories: daily (six to seven times a week), often (three to five times a week), and seldom (zero to two times a week). Academic performance was divided into two outcomes based on mean aggregate marks: good (≥65%) and poor (<65%).

Data collection procedure

Data was collected by the researchers themselves using a finalized self-administered questionnaire. Students were approached in their respective departments, and the questionnaire’s content and the purpose of the study were explained to them. The questionnaire was administered in the supervised classrooms, ensuring independent completion and preventing discussion among participants. Height was measured using a wall-mounted scale with participants standing still, legs straight, arms at their sides, line of sight parallel to the floor, and heels touching the flat surface. Weight was measured with a calibrated digital weight machine. Participants were instructed to take off their shoes and place aside any other accessories (e.g., cell phone, wallet, purse). Data of the last exams were obtained from the university’s examination branch, and all the personal identifiers were removed before data analysis.

Statistical analysis

Data were entered and analyzed using GraphPad Prism, version 9.5 (GraphPad Software, San Diego, CA, USA). Chi-square (χ2) test was applied to assess the association between academic performance and sociodemographic, socioeconomic, and nutritional factors. Binary logistic regression was performed to examine predictors of academic performance, and odds ratios (OR) with their 95% confidence interval (CI) estimates were computed.

Variables with p-values <0.05 were included in the final multivariate model. Model fit for multiple logistic regression was assessed with the Hosmer-Lemeshow test, McFadden's R^2^, and receiver operating characteristic curve (ROC-AUC). Predictive accuracy was evaluated with classification rates and ROC-AUC. Adjusted odds ratios (AOR) with their 95% CI estimates were reported. A 95% confidence level was used for analysis, and the threshold of significance was set at p-value <0.05.

Ethical approval

This study was conducted following approval from the Ethical Review Committee, Institute of Public Health, PUMHSW (Reference No. PUMHSW/SBA/CHS/170 dated March 20, 2023). Students meeting the eligibility criteria were informed about the study's purpose, and written consent was obtained. Information provided by the participants was kept strictly confidential, and all personal identifiers were removed prior to analysis.

## Results

Half of the sample comprised Bachelor of Medicine, Bachelor of Surgery (MBBS) students (50.5%). The mean age of participants was 21.5±1.5 years, with nearly half aged 22-23 years. Most students were day scholars (76.0%) and rural residents (62.5%). The mean BMI was 21.8±5.7 kg/m², and 50.0% students had a normal weight. Overall, 76.8% demonstrated good academic performance, with mean aggregate marks of 69.5±9.5 (Table 1).

**Table 1:** Baseline characteristics of the study participants (N=400) n: number of students; %: column percentage of grand total; MBBS: Bachelor of Medicine, Bachelor of Surgery; DPT: Doctor of Physical Therapy; BSPH: Bachelor of Science in Public Health; BSN: Bachelor of Science in Nursing; Pharm-D: Doctor of Pharmacy. Nutritional status assessed with body mass index (BMI) and classified as: underweight (<18.5 kg/m²), normal (18.5–24.9 kg/m²), and overweight (≥25.0 kg/m²). Good academic performance defined as mean aggregate marks ≥65% in the last exams.

Variables	Categories	n (%)
Age	≤19 years	41 (10.3)
	20 years	65 (16.3)
	21 years	90 (22.5)
	22 years	95 (23.8)
	≥23 years	109 (27.3)
Residence	Rural	150 (37.5)
	Urban	250 (62.5)
Accommodation	Hostler	96 (24.0)
	Day scholar	304 (76.0)
Field of study	MBBS	202 (50.5)
	DPT	54 (13.5)
	BSPH	51 (12.8)
	BSN	48 (12.0)
	Pharm-D	45 (11.3)
Nutritional status	Underweight	117 (29.3)
	Normal weight	200 (50.0)
	Overweight	83 (20.8)
Academic performance	Good	307 (76.8)
	Bad	93 (23.3)

Academic performance revealed significant association with field and year of the study. MBBS students (OR=6.23, 95% CI: 3-13.03, p<0.001) and Bachelor of Science in Public Health (BSPH) students (OR=2.91, 95% CI: 1.21-7.27, p<0.05) had higher odds of good academic performance. Fourth year students (OR=2.84, 95% CI: 1.54-5.37, p<0.05) and fifth year students (OR=7.15, 95% CI: 2.9-21.7, p<0.001) also performed significantly better. No significant associations were observed for age, residence, and accommodation (Table 2).

**Table 2:** Sociodemographic predictors of academic performance (N=400) n: number of students; %: row percentage; χ2: chi-square test statistic; OR: odds ratio, calculated from binary logistic regression; CI: confidence interval; Ref: reference category; MBBS: Bachelor of Medicine, Bachelor of Surgery; DPT: Doctor of Physical Therapy; BSPH: Bachelor of Science in Public Health; BSN: Bachelor of Science in Nursing; Pharm-D: Doctor of Pharmacy; *p<0.05, **p<0.001.

Variables	Categories	Good academic performance, n (%)	χ^2^ (p-value)	OR (95% CI)	p-value
Age	≤19 years	31 (75.6)	5.16 (0.271)	Ref	-
	20 years	44 (67.7)		0.68 (0.27-1.61)	0.384
	21 years	73 (81.3)		1.39 (0.56-3.33)	0.471
	22 years	71 (74.7)		0.95 (0.39-2.19)	0.914
	≥23 years	88 (80.7)		1.35 (0.56-3.13)	0.491
Residence	Rural	108 (72.0)	3.04 (0.82)	Ref	-
	Urban	199 (79.6)		1.52 (0.95-2.43)	0.083
Accommodation	Hostler	71 (74.0)	0.55 (0.458)	Ref	-
	Day Scholar	236 (77.6)		1.22 (0.71-2.06)	0.458
Field of study	Pharm-D	25 (55.6)	41.1 (< 0.05*)	Ref	-
	BSN	27 (56.3)		1.03 (0.45-2.34)	0.947
	DPT	36 (66.7)		1.6 (0.71-3.7)	0.259
	BSPH	40 (78.4)		2.91 (1.21-7.27)	<0.05*
	MBBS	179 (88.6)		6.23 (3-13.03)	<0.001**
Year of study	Second year	74 (63.8)	23.91 (<0.05*)	Ref	-
	Third year	75 (73.5)		1.58 (0.89-2.84)	0.124
	Fourth year	95 (83.3)		2.84 (1.54-5.37)	<0.05*
	Fifth year	63 (92.6)		7.15 (2.9-21.7)	<0.001**

Parental income was significantly associated with academic performance. Upper middle-class students (OR=2.17, 95% CI: 1.08-4.32, p<0.05) had better odds than lower-class students. Parental education and occupation showed slight variations in performance but failed to achieve significance (Table 3).

**Table 3:** Socioeconomic predictors of academic performance (N=400) n: number of students; %: row percentage; χ2: chi-square test statistic; OR: odds ratio, calculated from binary logistic regression; CI: confidence interval; Ref: reference category; *p<0.05, **p<0.001. Parental education defined as years of formal education and categorized into: illiterate (no formal education), primary (one to five years), secondary and higher secondary (six to 12 years), and graduation (≥14 years). Parental income defined as monthly income and categorized into: lower class (<50,000 PKR), lower-middle class (50,000-100,000 PKR), upper-middle class (100,001-200,000 PKR), and upper class (>200,000 PKR).

Variables	Categories	Good academic performance, n (%)	χ^2^ (p-value)	OR (95% CI)	p-value
Father’s occupation	Private employee	34 (69.4)	2.29 (0.807)	Ref	-
	Doctor	22 (75.9)		1.39 (0.5-4.13)	0.540
	Government employee	89 (76.1)		1.40 (0.66-2.92)	0.371
	Businessman	67 (79.8)		1.74 (0.77-3.91)	0.179
	Teacher	44 (80.0)		1.77 (0.72-4.42)	0.215
	Others	51 (77.3)		1.5 (0.65-3.49)	0.342
Mother’s occupation	Unemployed	252 (75.9)	0.78 (0.376)	Ref	-
	Employed	55 (80.9)		1.34 (0.72-2.68)	0.377
Father’s education	Illiterate	2 (40.0)	4.92 (0.178)	Ref	-
	Primary	10 (66.7)		3 (0.38-29.16)	0.302
	Secondary & higher secondary	88 (76.5)		4.89 (0.77-38.6)	0.091
	Graduation	207 (78.1)		5.35 (0.87-41.36)	0.069
Mother’s education	Illiterate	19 (67.9)	14.21 (0.136)	Ref	-
	Primary	40 (67.8)		1 (0.37-2.58)	0.995
	Secondary & higher secondary	147 (77.8)		1.66 (0.67-3.85)	0.251
	Graduation	101 (81.5)		2.08 (0.81-5.11)	0.116
Parental income	Lower class	35 (64.8)	23.66 (<0.05)	Ref	-
	Lower middle class	130 (76.5)		1.76 (0.90-3.40)	0.092
	Upper middle class	116 (80.0)		2.17 (1.08-4.33)	<0.05*
	Upper class	26 (83.9)		2.82 (0.99-9.4)	0.066

Among the nutritional factors, academic performance was significantly associated with mixed diet (OR=2.93, 95% CI: 1.78-4.81, p<0.001), three or more meals per day (OR=3.34, 95% CI: 1.14-9.41, p<0.05) and daily breakfast (OR=3.88, 95% CI: 2.02-7.44, p<0.001). Students with normal weight performed slightly better, but the differences were not statistically significant (Table 4).

**Table 4:** Nutritional predictors of academic performance (N=400) n: number of students; %: row percentage; χ2: chi-square test statistic; OR: odds ratio, calculated from binary logistic regression; CI: confidence interval; Ref: reference category; *p<0.05, **p<0.001. Frequency of breakfast, lunch, and dinner defined as the number of times each meal consumed per week and classified into three categories: daily (six to seven times a week), often (three to five times a week), and seldom (zero to two times a week).

Variables	Categories	Good academic performance, n (%)	χ^2^ (p-value)	OR (95% CI)	p-value
Nutritional status	Overweight	60 (72.3)	1.27 (0.530)	Ref	-
	Normal weight	157 (78.5)		1.4 (0.77-2.50)	0.262
	Underweight	90 (76.9)		1.28 (0.67-2.44)	0.457
Diet type	Veg	69 (62.2)	18.18 (<0.05*)	Ref	-
	Non-veg	12 (75.0)		1.83 (0.59-6.86)	0.323
	Mixed	226 (82.8)		2.93 (1.78-4.81)	<0.001**
Meals per day	One	9 (56.3)	9.07 (<0.05*)	Ref	-
	Two	92 (70.8)		1.88 (0.63-5.42)	0.240
	Three or more	206 (81.1)		3.34 (1.14-9.41)	<0.05*
Breakfast frequency	Often	31 (57.4)	18.85 (<0.05*)	Ref	-
	Seldom	93 (72.7)		1.97 (1.01-3.84)	<0.05*
	Daily	183 (83.9)		3.88 (2.02-7.44)	<0.001**
Lunch frequency	Often	16 (66.7)	1.68 (0.431)	Ref	-
	Seldom	45 (75.0)		1.5 (0.51-4.17)	0.440
	Daily	246 (77.8)		1.76 (0.69-4.17)	0.214
Dinner frequency	Often	4 (66.7)	0.39 (0.823)	Ref	-
	Seldom	15 (75.0)		1.5 (0.17-10.5)	0.688
	Daily	288 (77.0)		1.67 (0.23-8.72)	0.555

The multiple logistic regression model included the variables showing a significant association in binary logistic regression. The significant independent predictors of academic performance were being an MBBS student (AOR=6.08, 95% CI: 1.90-20.9, p<0.05), and fourth- (3.59, 95% CI: 1.81-7.37, p<0.05) and fifth-year student (7.93, 95% CI: 2.9-26.11, p<0.001). Other variables included in the model including, breakfast frequency, meals per day, diet type, and parental income were not statistically significant but showed detectable trends (Table 5).

**Table 5:** Multiple logistic regression analysis of predictors of academic performance (N=400) MBBS: Bachelor of Medicine, Bachelor of Surgery; DPT: Doctor of Physical Therapy; BSPH: Bachelor of Science in Public Health; BSN: Bachelor of Science in Nursing; Pharm-D: Doctor of Pharmacy; AOR: adjusted odds ratio, calculated from binary logistic regression for variables with p<0.05 on binary logistic regression; CI: confidence interval; Ref: reference category; *p<0.05, **p<0.001.

Variables	Categories	AOR (95% CI)	p-value
Field of study	Pharm-D	Ref	-
	BSN	1.51 (0.6-3.84)	3.380
	DPT	1.41 (0.42-4.89)	0.582
	BSPH	3.01 (0.8-12.07)	0.110
	MBBS	6.08 (1.9-20.9)	<0.05*
Parental income	Lower class	Ref	-
	Lower middle class	0.94 (0.42-2.05)	0.886
	Upper middle class	0.79 (0.33-1.83)	0.591
	Upper class	1.31 (0.38-5.03)	0.675
Meals per day	One	Ref	-
	Two	1.59 (0.37-6.19)	0.513
	Three or more	2.28 (0.63-7.65)	0.191
Year of study	Second year	Ref	-
	Third year	1.96 (1.03-3.81)	0.0527
	Fourth year	3.59 (1.81-7.37)	<0.05*
	Fifth year	7.93 (2.9-26.11)	<0.05**
Diet type	Veg	Ref	-
	Non-veg	1.02 (0.24-4.91)	0.977
	Mixed	0.86 (0.31-2.18)	0.759
Breakfast frequency	Often	Ref	-
	Seldom	1.37 (0.59-3.16)	0.458
	Daily	2 (0.7-5.47)	0.184

The model demonstrated a good fit and acceptable discrimination (AUC=0.77). The model correctly classified 79.3% of cases, with a positive predictive value of 80.8% and a negative predictive value of 63.9% (Table 6).

**Table 6:** Summary of the model fit and diagnostic AICc: Akaike Information Criterion corrected; ROC AUC: receiver operating curve area under curve.

Metric	Value
AICc	397.3
McFadden’s R^2^	0.17
Hosmer-Lemeshow test	χ^2^ = 4.07, p = 0.851
ROC AUC	0.77
Correctly classified	79.3%
Positive predictive value	80.8%
Negative predictive value	63.9%

## Discussion

This study analyzed the sociodemographic, socioeconomic, and nutritional predictors of academic performance among 400 female undergraduate medical and health sciences students. Field and year of study, parental income, diet type, meals per day, and daily breakfast significantly impacted the academic outcomes, whereas year and field of study remained independent predictors in the multivariate model.

Sociodemographic factors are well-known determinants of academic achievement [13]. MBBS students consistently secured higher marks compared to their allied counterparts. These results are aligned with the studies from Riyadh, Saudi Arabia, and Lahore, Pakistan, where MBBS students outperformed the allied students [13,14]. Generally, the MBBS discipline is well-structured, skill-demanding, with rigorous and competitive admission criteria, which may explain the academic superiority over allied fields. Similarly, performance gradually increased with advancing years, suggesting adaptation to workload, refined learning techniques, and improved ability to understand the course structure and examination pattern over time. Although age showed no significant impact, older students performed better, reflecting accumulated academic experience and curriculum adoption. Accommodation and residence did not demonstrate a statistical association in this study. Despite previous studies noting better performance in hostlers [15,16], day scholars performed slightly better in our study. This difference may be due to unequal sample distribution and external factors.

Parental income revealed a significant positive relationship with academic outcome; students from the upper and upper-middle classes are more likely to perform better, aligning with previous studies from Pakistan and Sudan [6,17]. Higher socioeconomic status is linked to a better learning environment, academic support, and access to resources. While students with professional fathers and employed mothers showed slightly better academic outcomes, these results failed to achieve statistical significance. This implies that parental occupation may provide better opportunities for learning and growth, but may not independently result in good academics. Parental education showed a clear but non-significant upward trend, with graduate parents linked to higher student performance. These results are consistent with reported literature from the United States of America (USA) [18]. The lack of significance in our study may be explained by varying definitions, assessment methods, and scales of education throughout the world.

Nutritional status is a fundamental predictor of human growth and efficiency. Studies conducted on medical and health sciences students from Saudi Arabia and Turkey have shown associations between nutritional status and higher grade point average (GPA) [14,19]. In our study, students with normal weight performed academically better than those who were overweight or underweight; however, the difference was not significant. These variations in results can be attributed to the difference in outcome assessment, methodologies, and sampling fluctuations. Further longitudinal studies are recommended to understand the potential relationship and implement the required nutritional policies for students. Among nutritional factors, mixed diet, three or more meals per day, and daily breakfast were significantly associated with academic performance. Data regarding these dietary factors is lacking in the reported literature for university students, but studies on school students in Iraq, Indonesia, and Malaysia have repeatedly recognized daily breakfast and a balanced diet as significant predictors of academic excellence [20-22].

In the multivariate analysis, year of study and field of study emerged as significant independent predictors of good academic performance. Nutritional factors, dietary patterns, and socioeconomic status showed a higher strength of association in binary logistic analysis, but did not retain statistical significance in the multivariate model. This suggests academic-related variables have a strong impact on academic performance, while nutritional and socioeconomic variables may affect it indirectly. The overall model demonstrated satisfactory predictive accuracy and acceptable discriminatory power and can be used to detect at-risk students.

Limitations of the study

This study did not use any popular nutritional and socioeconomic scale due to difficulty in its use and unfamiliarity with the study participants. Academic performance was only determined by mean aggregate marks, and it didn’t account for other assessment methods, including assignments, projects, comprehensive tests, and class performance. The use of self-reported data for selected variables may introduce minimal reporting bias; however, the key outcome variables were objectively measured. The model has limited explanatory power, a lower negative predictive value due to insufficient sample size in specific subgroups, and potential multicollinearity.

## Conclusions

Academic performance is multifaceted and is influenced by academic-related factors, particularly academic progression and the field of study. Nutritional and socioeconomic factors had a limited independent effect. Structured curriculum, cumulative academic exposure, and intensive coursework play a central role in determining student achievement, while nutritional and socioeconomic factors may offer a supportive effect.

Targeted academic support and healthy lifestyle promotion, by incorporating nutritional awareness and student wellness initiatives, within university settings, may enhance student outcomes. Future research should employ standardized nutritional and socioeconomic assessment tools and integrate advanced analytical approaches, including machine learning algorithms and neural network models, to enhance predictive accuracy|.

## Appendices

Appendix A: Questionnaire

ID# …………………….

Roll no: …………………….

Date: ………. /………./ ………………

Section-A Sociodemographic characteristics

1. Age      …………… years

2. Residence      (  ) Urban      (  ) Rural

3. Accommodation      (  ) Home      (  ) Hostel

4. Field of study      (  ) MBBS      (  ) BSPH      (  ) DPT      (  ) Pharm-D      (  ) BSN

5. Year of study      (  ) 1st year      (  ) 2nd year      (  ) 3rd year      (  ) 4th year      (  ) 5th year

Section-B Socioeconomic status

1. Father’s occupation      ………………………………………..

2. Mother’s employment status      (  ) Employed      (  ) Unemployed

3. Father’s Education

      (  ) Illiterate (0 years of formal education)

      (  ) Primary (1 - 5 years)

      (  ) Secondary and higher secondary (5 -12 years)

      (  ) Graduation (≥ 14 years)

4. Mother’s Education

      (  ) Illiterate (0 years of formal education)

      (  ) Primary (1 - 5 years)

      (  ) Secondary and higher secondary (5 -12 years)

      (  ) Graduation (≥ 14 years)

5. Parental Income

      (  ) Lower class (< 50,000 rupees per month)

      (  ) Lower middle class (50,000 - 100,000 rupees per month)

      (  ) Upper middle class (100,001 - 200,000 rupees per month)

      (  ) Upper class (> 200,000 rupees per month)

Section-C Nutritional information

1. Height      …………… m

2. Weight     …………… kg

3. BMI      …………… kg/m2

4. Nutritional status      (  ) Overweight      (  ) Normal weight      (  ) Underweight

5. Diet type      (  ) Veg      (  ) Non-veg      (  ) Mixed

6. Number of meals per day      (  ) One      (  ) Two      (  ) Three or more

7. Breakfast frequency     

      (  ) Daily (6 to 7 times a week)     

      (  ) Often (3 to 5 times a week)

      (  ) Seldom (0 to 2 times a week)

8. Lunch frequency

      (  ) Daily (6 to 7 times a week)

      (  ) Often (3 to 5 times a week)

      (  ) Seldom (0 to 2 times a week)

9. Dinner frequency

      (  ) Daily (6 to 7 times a week)

      (  ) Often (3 to 5 times a week)

      (  ) Seldom (0 to 2 times a week)

Section-D Academic information

1. Marks obtained in last exams      ……………

2. Total marks      ……………

3. Mean aggregate marks      ……………

Credit: Created by the authors for this study.
